# Molecular Correlation between Larval, Deutonymph and Adult Stages of the Water Mite *Arrenurus (Micruracarus) Novus*

**DOI:** 10.3390/life10070108

**Published:** 2020-07-09

**Authors:** Pedro María Alarcón-Elbal, Ricardo García-Jiménez, María Luisa Peláez, Jose Luis Horreo, Antonio G. Valdecasas

**Affiliations:** 1 Instituto de Medicina Tropical & Salud Global (IMTSAG), Universidad Iberoamericana (UNIBE), 22333 Santo Domingo, Dominican Republic; Pedro.Alarcon@uv.es; 2 Museo Nacional de Ciencias Naturales, CSIC. C/José Gutiérrez Abascal, 2, 28006 Madrid, Spain; rgarcia@mncn.csic.es (R.G.-J.); maaller@yahoo.es (M.L.P.); valdeca@mncn.csic.es (A.G.V.); 3 UMIB Research Unit of Biodiversity (UO, CSIC, PA), C/Gonzalo Gutiérrez de Quirós s/n, 33600 Mieres, Spain

**Keywords:** Acari Actinotrichida, COI, cytochrome B, genetic identification, Hydrachnidia, Culicidae, reverse taxonomy, species identification

## Abstract

The systematics of many groups of organisms has been based on the adult stage. Morphological transformations that occur during development from the embryonic to the adult stage make it difficult (or impossible) to identify a juvenile (larval) stage in some species. Hydrachnidia (Acari, Actinotrichida, which inhabit mainly continental waters) are characterized by three main active stages—larval, deutonymph and adult—with intermediate dormant stages. Deutonymphs and adults may be identified through diagnostic morphological characters. Larvae that have not been tracked directly from a gravid female are difficult to identify to the species level. In this work, we compared the morphology of five water mite larvae and obtained the molecular sequences of that found on a pupa of the common mosquito *Culex* (*Culex*) *pipiens* with the sequences of 51 adults diagnosed as *Arrenurus* species and identified the undescribed larvae as *Arrenurus* *(Micruracarus) novus*. Further corroborating this finding, adult *A. novus* was found thriving in the same mosquito habitat. We established the identity of adult and deutonymph *A. novus* by morphology and by correlating COI and cytB sequences of the water mites at the larval, deutonymph and adult (both male and female) life stages in a particular case of ‘reverse taxonomy’. In addition, we constructed the Arrenuridae phylogeny based on mitochondrial DNA, which supports the idea that three *Arrenurus* subgenera are ‘natural’: *Arrenurus*, *Megaluracarus* and *Micruracarus*, and the somewhat arbitrary distinction of the species assigned to the subgenus *Truncaturus.*

## 1. Introduction

The systematics of many groups of organisms has traditionally been based on the adult stage [1]. For species showing a deep morphological transformation from the embryonic to the adult stage, due to a change in habitat or different behavior (parasitic and free-living stages) it may be difficult, if not impossible, to identify the juvenile stage without a previous correlation with the corresponding adult.

Members of the clade Hydrachnidia (Acari Actinotrichida) mainly inhabit continental waters [2] and are characterized by three main active life stages—larval, deutonymph and adult—with intermediate dormant stages [3]. Deutonymphs and adults share a characteristic morphology and may be identified through diagnostic characters. Larval morphology is very different from that of the two other stages. The standard procedure to match the larvae of a species with its corresponding adult is to track them directly from a fertilized female. In short, a fertilized female is kept in a small vial with a strip of paper or another substrate until they oviposit eggs and larvae emerge [4,5]. It is hard to identify the species level of larvae that have not been tracked in this way with certainty.

The water mite genus *Arrenurus* has a worldwide distribution, except for the perpetual snow regions, but there are no cosmopolitan species; rather, each main area upholds its own set of species. There are 152 *Arrenurus* in Europe and, of these, about 30% have a wide distribution [6,7,8]. As in other Hydrachnidia genera, *Arrenurus* deutonymphs and adults share diagnostic characters, however, the larval stage has a distinct morphology and requires independent morphological characterization. Water mite larvae usually attach to insects at preimaginal stages before they hatch, using adults as vector to disperse [9]. Adults and larvae of the same species, therefore, may be found in very different locations in their life cycle. Some *Arrenurus* species have occasionally been recorded as an ectoparasite of aquatic insects at the juvenile stage, either nymphs or larvae [2]. Some have even been recorded in insect exuviae [6,10]. Given these challenges, species identification of isolated *Arrenurus* larvae is fraught with uncertainty.

In this work, we characterized *Arrenurus* sp. larvae that were attached to larvae and a pupa of the common mosquito *Culex* (*Culex*) *pipiens* and cross-correlate their sequences with those of 51 adult *Arrenurus* species. One of these species, *Arrenurus* (*Micruracarus*) *novus* George, 1884, was in the same mosquito habitat from which the *Arrenurus* larvae were isolated. We compare cytochrome C oxidase subunit I (COI) and cytochrome B (cytB) DNA sequences of the water mite larvae with those of *A. novus* deutonymphs and adults (male and female) and other *Arrenurus* species, in order to establish the identity of the water mite larvae in a particular case of ‘reverse taxonomy’ [11].

## 2. Materials and Methods

### 2.1. Taxon Sampling

**Figure 1:**
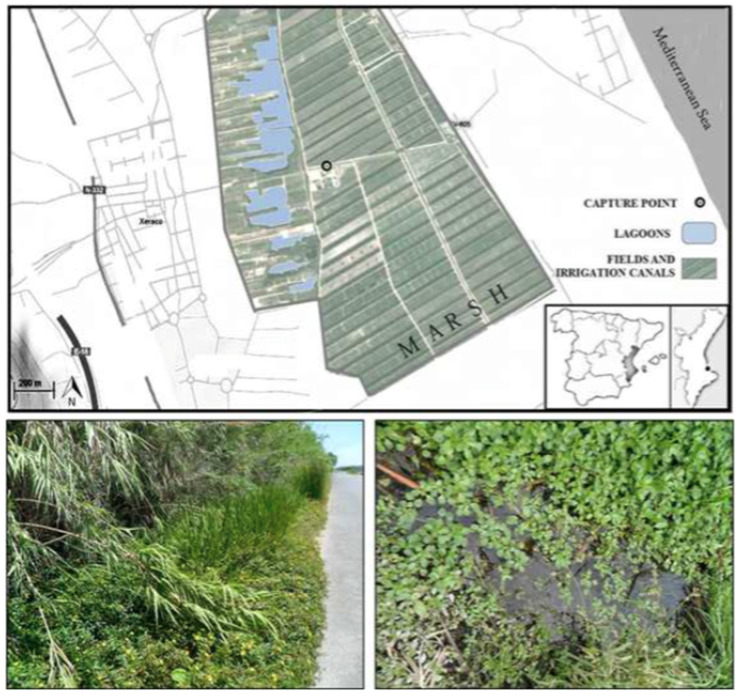
Map of the sampling site (Xeraco, Valencia, Spain).

Water mite and mosquito specimens were both collected during a survey of mosquitoes at preimaginal stages carried out every two weeks between May and October of 2012 at Acequia del Caminàs (UTM 30S 741855.92 E 4324431.48), a semiartificial irrigation channel within the marsh waters of the Xeraco and Xeresa wetlands (Valencia, Spain; Figure 1). For more details on the characteristics of the area, see Alarcón-Elbal et al. [12]. Further collection efforts were carried out in May 2019 without success.

We used a quick dipping technique [13] with a standard 500-mL mosquito dipper (BioQuip Products Inc., Rancho Dominguez, CA, USA). Culicids at preimaginal stages were sorted manually at the laboratory and then transferred to a 60 °C water bath for a minute and then to absolute ethanol. Culicid specimens with ectoparasites and free-living water mites in the same sample were both preserved in absolute ethanol and stored at −20 °C.

Preservation in absolute ethanol ensures that both microscopic and molecular techniques can be used on any water mite specimen collected from potential hosts or directly from the field [14,15]. Both the culicids and the water mite specimens were morphologically identified, and subsequently used for DNA extraction.

### 2.2. Morphological Identification of Culicids

Morphological identification of culicids at preimaginal stages were made to the species level based on external morphology and diagnostic characters related with chaetotaxy, as described by Schaffner et al. [16]. Specimens were examined under a Nikon SMZ-1B Stereozoom microscope.

### 2.3. Morphological Identification of Water Mites

#### 2.3.1. Rearing of Adult Female Water Mites

In an attempt to rear larvae, six live adult *Arrenurus* sp. females were collected in the field and then kept under laboratory conditions in individual transparent poliestirene plastic containers (diameter 10–15 mm, height 15 mm) with a piece of paper and mineral water at room temperature for 14–15 days [4]. Following the monitoring period, the females were preserved in absolute ethanol for morphological identification of the species level.

#### 2.3.2. Morphological Identification of Water Mite Adults, Deutonymphs and Larvae

Morphological identification of the adult and deutonymph water mite specimens collected from the field site was performed using the diagnostic characters originally described by George [17] and subsequently revised by Viets [18] and Gerecke [2].

The study of larval water mite specimens was based on four specimens found anchored on the body of a culicid larva (L1–L4 in Table 1) and one larva found on a culicid pupa (L5). The culicid larva, pupa and attached water mite larvae were examined first under a Bausch and Lomb stereomicroscope and then with laser scanning confocal microscopy (LSCM, Leica TCS SPE). The host attachment site, where the water mite larval mouthparts were embedded, was recorded and the larvae were individually and carefully detached using fine tweezers and transferred to a drop of glycerol on a microscope slide for LSCM stack acquisition. After imaging, the five larvae were then processed for molecular analyses.

#### 2.3.3. Laser Scanning Confocal Microscopy Imaging

Laser scanning confocal microscopy (LSCM, Leica TCS SPE) was employed to acquire z-stack images with the following objectives: 10×/0.30 NA, 20×/0.70 NA, 40×/1.25 NA oil immersion and 63x/1.30 NA glycerol immersion. The samples were subjected to an excitation wavelength of 488 nm and an emission range between 520 and 660 nm. For further information on the stack acquisition procedure, see Valdecasas and Abad [14]. Serial images were processed with Amira (ver. 5.4.3) and ImageJ/Fiji to obtain maximum 2D projections and Voltex volume renderings. Different parameters of visualization were used with Voltex to best highlight the characters of interest. Fiji/ImageJ were used to take all measurements directly from the image stacks. As all the material was used for the subsequent molecular analyses, no morphological vouchers could be kept. Therefore, a photographic voucher comprised of the full set of original unaltered stacks is stored in the confocal collection at the National Museum of Natural Sciences of Spain (CSIC–MNCN).

### 2.4. Molecular Analyses

A total of nine water mite samples—three adults, one deutonymph and five larvae—were processed for molecular analyses. DNA extraction, PCR amplification and sequencing were performed as previously described) [15]. The COI gene was amplified using the primer pair LCO1490 and HC02198 [19]. For the larval water mite samples (*n* = 5), the internal primer COI-AV-F (5′-ATAAGATTTTGACTTCTYCC-3′) and HC02198 (360 basepairs length) were used because the complete amplification of the gene was not successful. CytB was amplified using the primer pair COB-F/R [20]. Sequencing reactions were performed using ABI BigDye^®^ v3.1 Cycle Sequencing Kit (Applied Biosystems) and run on an ABI 3100 Genetic Analyzer. Sequence chromatograms were checked for accuracy and edited using Sequencher^®^ version 5.0 (Gene Codes Corporation, Ann Arbor, MI, USA). DNA sequences were deposited in GenBank (accession numbers MT598102 to MT598106 for COI and MT607634 to MT607637 for cytB).

The sequences of both gene fragments were analyzed for all three life cycle stages in order to molecularly correlate the undescribed larvae with the diagnosed adults and deutonymphs. Genetic distances were estimated using the Kimura-2-Parameter (K2P) distance model [21], as implemented in MEGA v.7 [22].

Additionally, COI and cytB sequences of *Arrenurus* species were searched in GenBank (https://www.ncbi.nlm.nih.gov/genbank/; accessed 21 May 2020) in order to estimate the genetic distances among all available sequences, including the new ones of this study. The distances of the GenBank sequences were calculated using MEGA. A Maximum-Likelihood phylogenetic tree of COI sequences was constructed with the IQ-TREE web server (http://iqtree.cibiv.univie.ac.at) with the following settings: automatic detection of substitution model, perturbation strength of 0.5, IQ-TREE stopping rule of 100, ultrafast bootstrap analysis (1000 replicates) and SH-aLRT single branch tests (1000 replicates). For this tree, two outgroup sequences were used: *Torrenticola lundbladi* (GenBank accession number JX629050) and *Lebertia maderigena* (KX421869).

## 3. Results

### 3.1. Morphological Identification of Culicids

**Figure 2:**
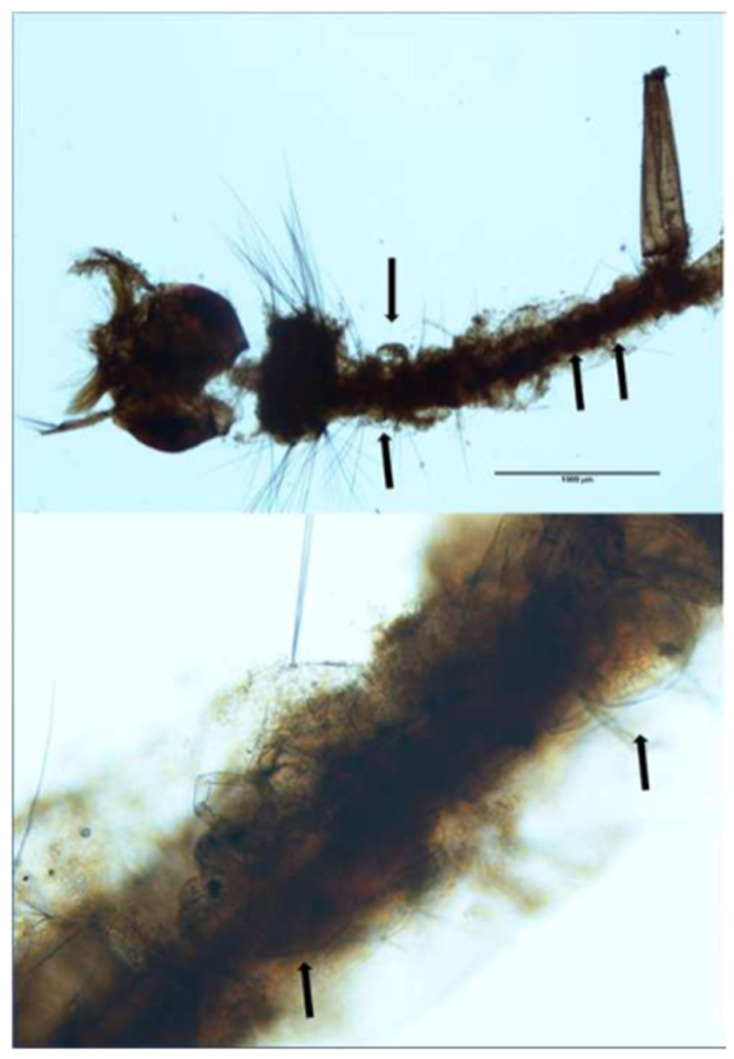
*Culex pipiens* larva with *Arrenurus* sp. larvae attached (arrows) and detail.

**Figure 3:**
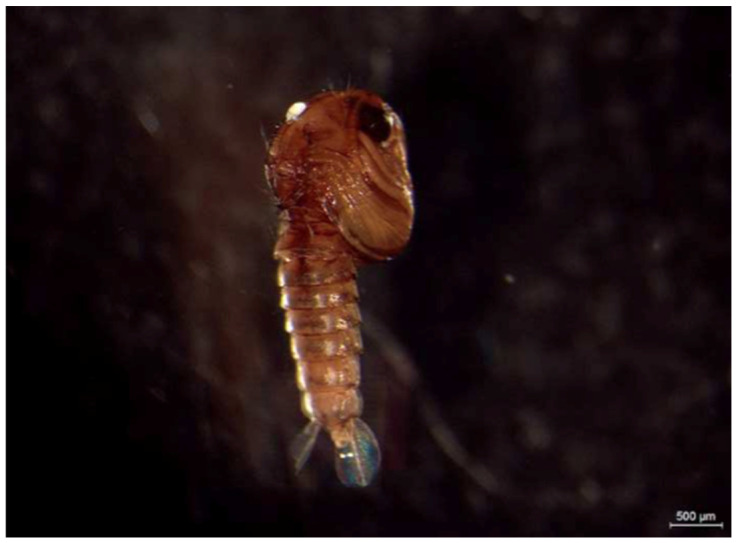
*Culex pipiens* pupa with *Arrenurus* sp. larva attached (white dot).

**Figure 4:**
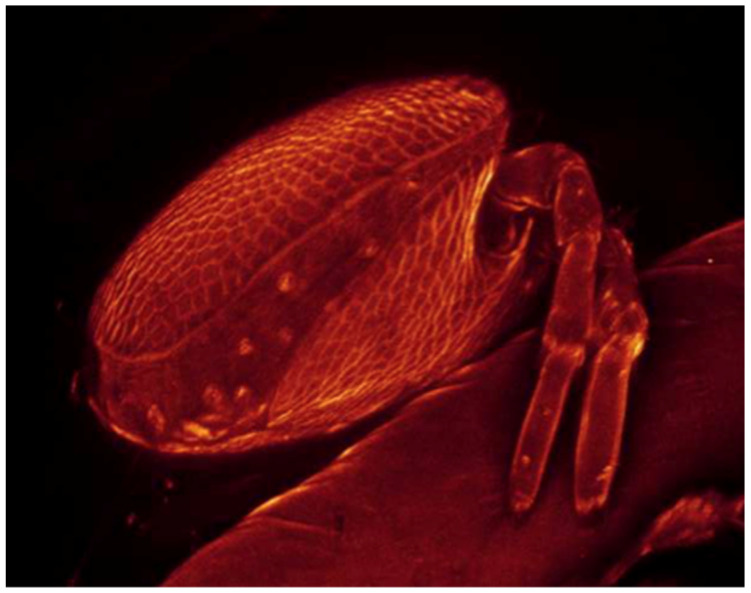
*Culex pipiens* pupa with *Arrenurus* sp. larva attached (laser scanning confocal microscopy (LSCM) image).

All of the culicid specimens were identified as *Culex* (*Culex*) *pipiens* Linnaeus, 1758. The principal and reliable characters for diagnosis at the larval stage are the syphon index, the syphon/saddle index, the branch number of seta one on abdominal segments III–IV, 1a–S tuft, 1b–S tuft and syphon shape [23]. One culicid larva and one pupa were parasitized with four and one water mite larvae, respectively (Figure 2, Figure 3 and Figure 4).

### 3.2. Morphological and Molecular Identification of Water Mites

#### 3.2.1. Rearing of Adult Female Water Mites

Six *Arrenurus* females were maintained for 14–15 days, but no eggs or larvae were observed during this period. We were not able to ascertain whether the females were gravid and if the lack of eggs and larvae was due to unsuitable laboratory conditions.

#### 3.2.2. Larval Description

**Figure 5:**
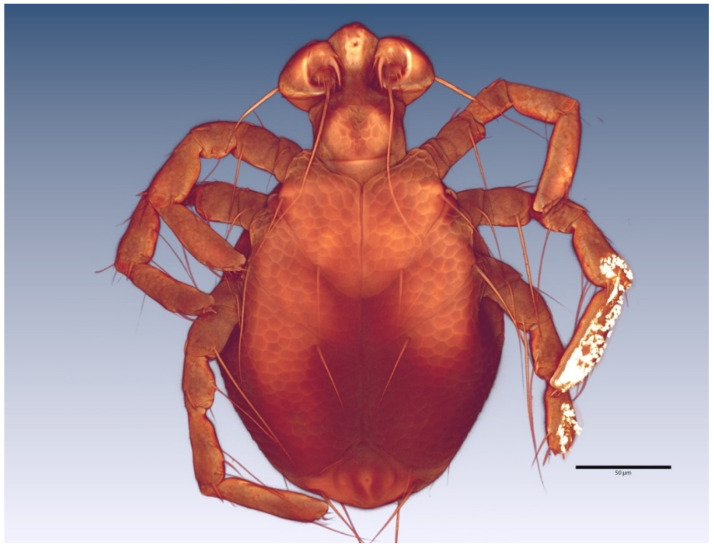
*Arrenurus (Micruracarus) novus* larva, ventral view (LSCM).

**Figure 6:**
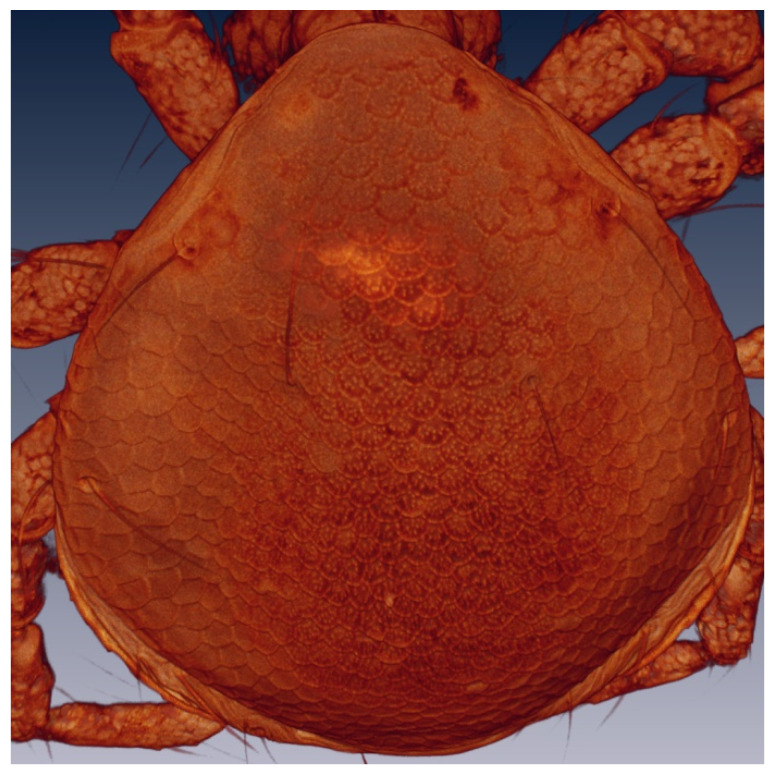
*Arrenurus (Micruracarus) novus* larva, dorsal view (LSCM).

**Figure 7:**
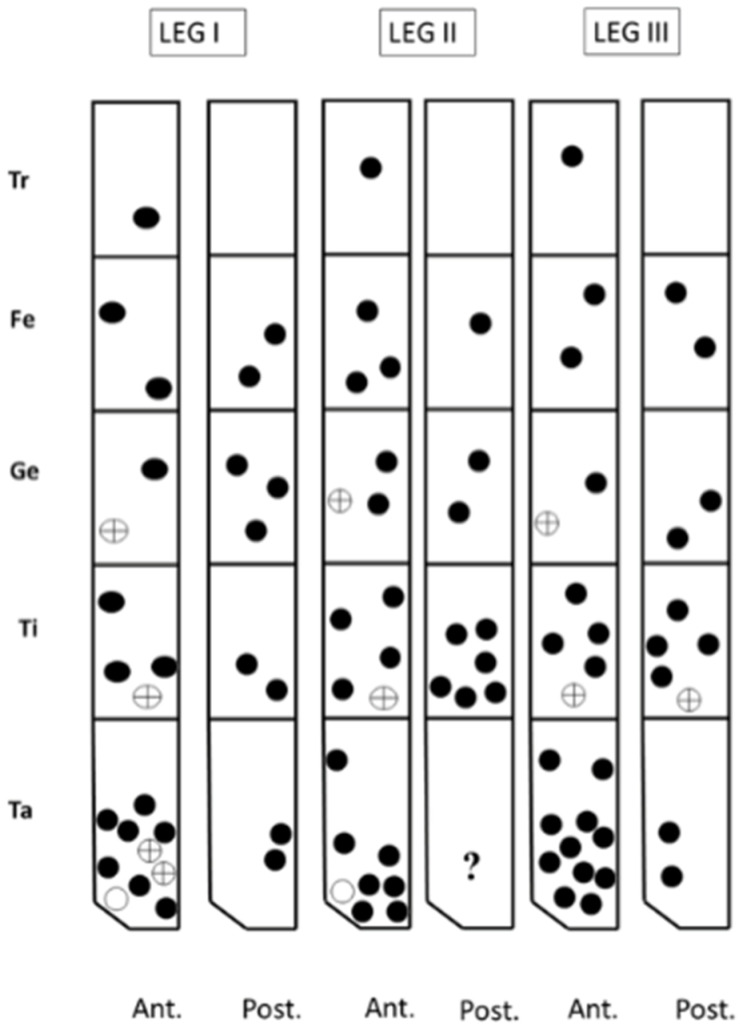
ASchematic showing the distribution of setae on the leg segments of an Arrenurus (Micruracarus) novus larva. Tr: trochanter; Fe: femur; Ge: genu; Ti: tibia; Ta: tarsus. 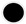 Round setae; 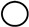 solenidum; 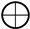 other setae.

The description of the water mite larvae is preliminary: confocal microscopy could not resolve some of the extremely fine morphological details, an issue faced by other researchers of water mite larvae [7]. Therefore, scanning electron microscopy analyses are necessary for a complete description. The terminology used here follows that of Zawal [7]. Figure 5 and Figure 6 show a Voltex projection of the ventral and the dorsal habitus of a larva, respectively. The projections do not indicate morphometric distances, and the scales are only an approximation of size. Table 1 and Table 2 include some standard morphometric data taken independently of the Voltex projections. Figure 7 shows a schematic of the approximate distribution of setae on the leg segments.

**Figure 8:**
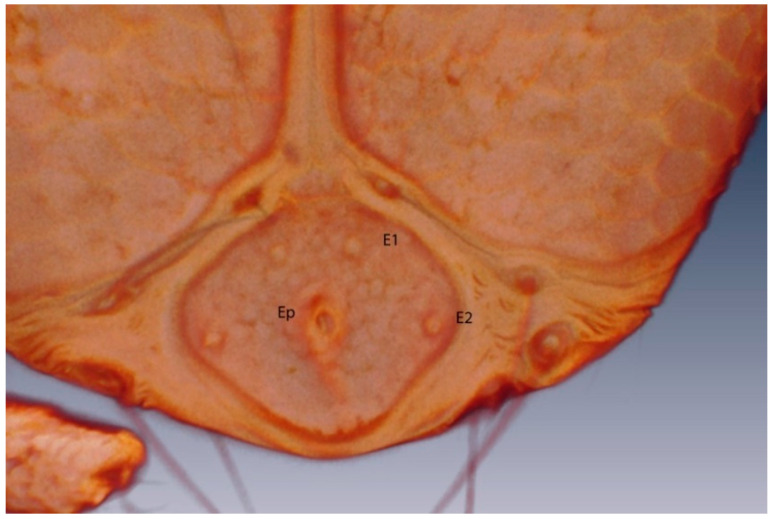
*Arrenurus (Micruracarus) novus* larva, excretory plate (LSCM).

*Arrenurus novus* larvae (see molecular analysis below) may be distinguished from other previously described *Micruracarus* larvae [7] by the following set of characters: dorsal plate ovoid, narrowed anteriorly; coxas distinct, ratio lateral length of coxal plate I, II and III: 5:3:4; a hexagonal excretory plate perimeter and the excretory pore in line or slightly above E2 setae (Figure 8). Total length of Leg1 < Leg II = Leg III.

**Figure 9:**
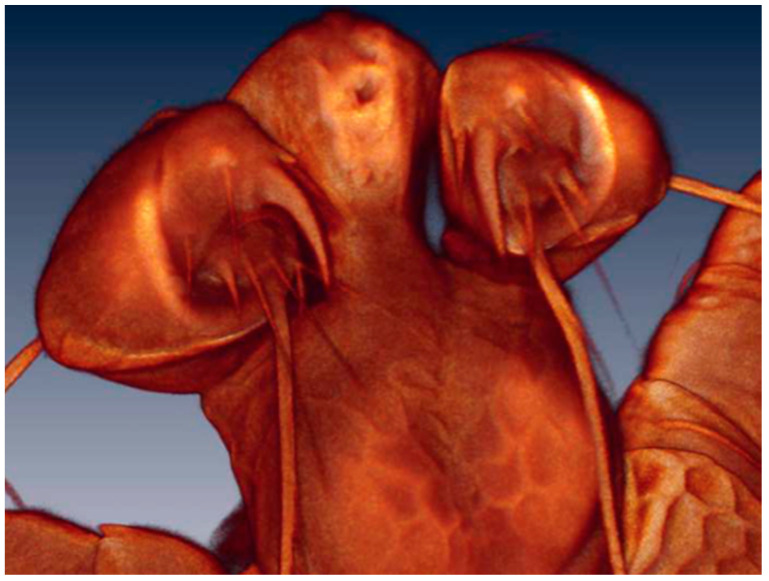
*Arrenurus (Micruracarus) novus* larva, pedipalp (LSCM).

The number of setae on the pedipalp segments are in agreement with Zawal’s description of *Arrenurus* larvae [7]: none on P-I; one on P-II; two on P-III, one long and thick and the other short; four on P-IV, three thin and one thick; and a solenoid and seven setae on P-V (Figure 9).

#### 3.2.3. Morphological Identification of Adult and Deutonymph Water Mites

**Figure 10:**
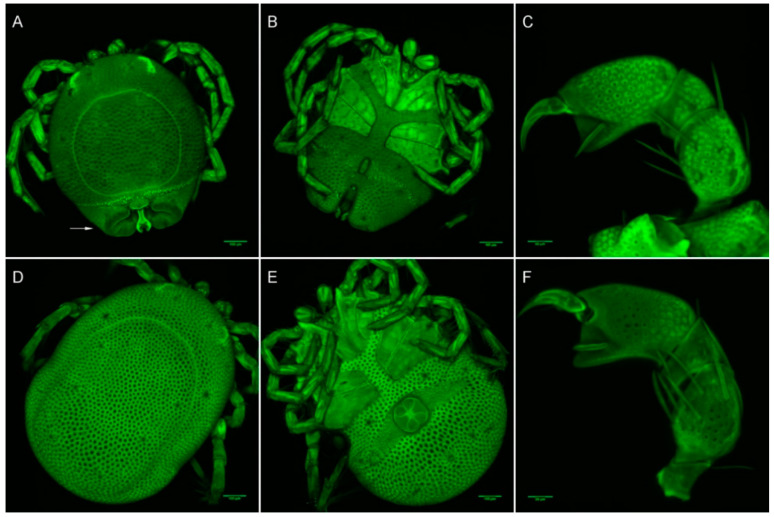
*Arrenurus (Micruracarus) novus* adults (LSCM). (**A**) male, dorsal; (**B**) male, ventral; (**C**) male, palp; (**D**) female, dorsal; (**E**) female, ventral; (**F**) female, palp.

**Figure 11:**
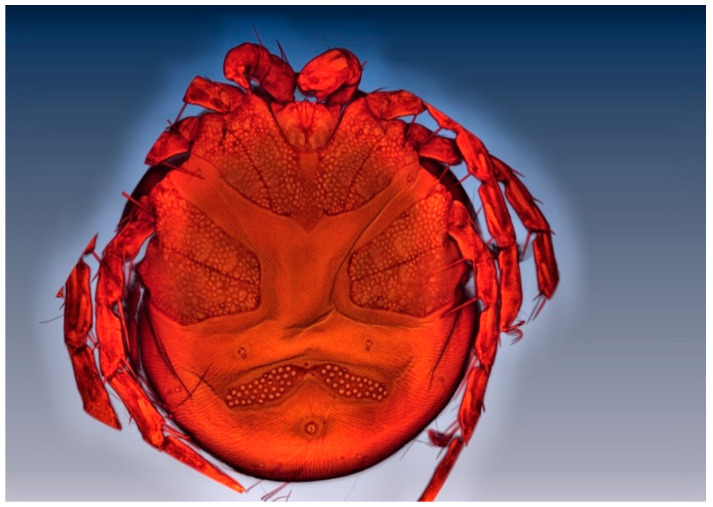
Ventral view of *Arrenurus (Micruracarus) novus* deutonymph.

Seven male and six female adults plus two deutonymphs were identified as *A. novus*, a species found in standing waters throughout Europe [2]. Among the main diagnostic characters identifying *A. novus* adults are the petiole in the male (see arrow in Figure 10A), the setation of male and female palps, and the shape and length of cauda (Figure 10). The deutonymph diagnosis was based on palp shape and setation and habitat co-occurrence (Figure 11): approximate body length: 526 µm; body width: 467 µm; P-1 without setae; P-II with three medial setae; P-III with one medial setae; PIV uncate and a strong distal setae and PV with a basal fine setae.

### 3.3. Molecular Analyses

**Figure 12:**
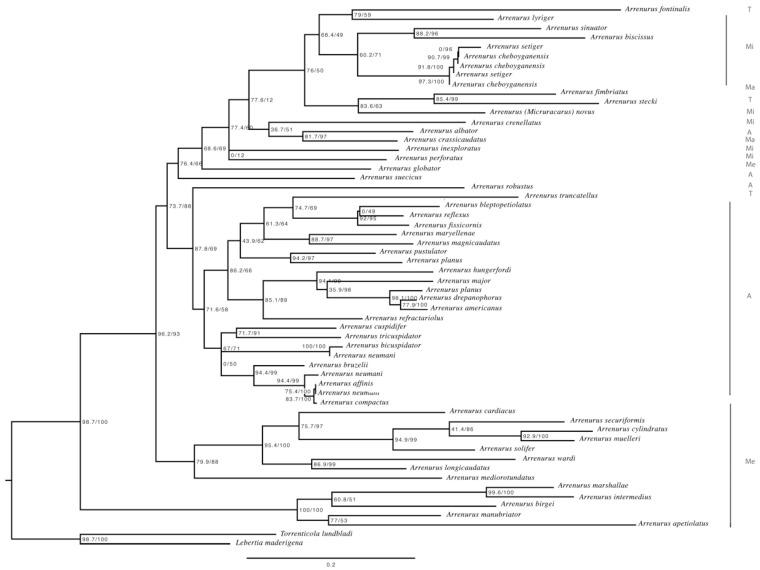
Maximum Likelihood phylogenetic tree of the *Arrenurus* C oxidase subunit I (COI) sequences employed in this study and the outgroups (*Torrenticola lundbladi* and *Lebertia maderigena*). Node numbers show SH-aLRT (left) and bootstrap (right) support values (1000 replicates). Letters at right show subgenus acronyms: *Arrenurus* (A), *Micruracarus* (Mi), *Truncaturus* (T), *Megaluracarus* (Me) and *Micrarrenurus* (Ma).

GenBank identified 990 *Arrenurus* barcoding COI sequences but no *Arrenurus* cytB sequences (probably because cytB is not generally used for species delimitation). These sequences belonged to 51 species of the genus plus *A. novus*, whose sequence is reported here for the first time (genetic distances among species are shown in Table S1 and phylogenetic tree in Figure 12). The mean genetic distances among the 52 *Arrenurus* species was 16.4% (standard deviation = 4.1). The here-amplified COI sequences from all the different *A. novus* life-cycle stages were all the same.

Very few phylogenetic information about Arrenurus currently exist [24]. The here-constructed COI phylogenetic tree (Figure 12, Material S1) may be divided into three main arms: the upper branch is dominated by species belonging to the subgenus *Micruracarus*, with occasional incursions of species belonging to the subgenera *Arrenurus*, *Truncaturus*, *Megaluracarus* and *Micrarrenurus*. In this upper branch is located *Arrenurus* (*Micruracarus*) *novus.* The intermediate branch of the tree is composed exclusively of species of the subgenus *Arrenurus*, except for a sequence belonging to subgenus *Truncaturus*. The lower section of the tree consists exclusively of species belonging to the sub-genus *Megaluracarus*, with European and American representatives. 

The 360-bp COI fragment was successfully amplified only from one larvae; however, no genetic variation was found among the sequences of this region (two adults, one deutonymph and one larvae). Genetic differences were, however, observed in the 330-bp cytB fragment (Table 3). One of the adults and all five larvae shared the same cytB haplotype, whereas the deutonymph and the other adult shared a different haplotype (Table 3).

## 4. Discussion

The number of molecular sequences available for Hydrachnidia has increased substantially in recent years. GenBank list 6450 Hydrachnidia nucleotide sequences (as of 21 May 2020). We did not search the BOLD Systems database for two main reasons: many of the *Arrenurus* sequences are privately held, and it was recently shown that some water mite sequences do not agree with their species assignment [25]. Of the GenBank sequences, almost 70% are identified to the generic level. Some new species have been described with morphological and molecular data [15,26]. For the genus *Arrenurus,* there are 1071 sequences (990 of them belonging to COI gene) but only 198, representing 51 taxa, are at the species level. These were employed to the genetic distance calculations. All of the *Arrenurus* sequences in GenBank identified to the species level are based on the adult stage; sequences obtained from larvae are only given a generic rank and are frequently singled out as operational taxonomic units (e.g. [27]). There is a need for more species-level sequences based on different life cycle stages to widen the usefulness of this type of data to address ecological, biogeographical and evolutionary questions. We identified the adult and deutonymph water mite specimens as *A. novus*, a broadly distributed species found from the Western Palearctic to the Afrotropic, commonly in standing waters [2,25]. However, according to Gerecke et al. [2], it has been “little reported in Europe”.

In the present study, we combined morphological and molecular techniques to associate undescribed larvae with corresponding adults and deutonymphs for the first time in water mites. Associations among life cycle stages are especially relevant for this genus given that less than 20% of arrenurid larvae are known [28]. To link undescribed larval specimens with other life stages (i.e., deutonymph and adult), we employed a DNA-based identification method in which sequences are considered as standardized comparative characters that can be used to support and integrate morphological datasets [29]. Even in cases with very small genetic distances, these data can be useful for species identification [30]. The results of our analyses of two mitochondrial genes (COI and cytB), which proved to be informative, clearly support the same conclusion: the larvae belong to the species *A. novus*. The larval COI sequences were compared with 52 adult *Arrenurus* species sequences (51 were from GenBank; the *A. novus* sequences, from one adult male, two adult females, one deutonymphs and one larvae, are provided for the first time here). Genetic distances among the three *A. novus* life-cycle stages were 0.000 (the same DNA sequence), confirming the co-specificity of the larvae, deutonymph and adults. The distance of *A. novus* with other *Arrenurus* species ranged between 9.8 with *A. setiger* and 24.3 with *A. megalurus* (see their phylogenetic relationships in Figure S1). The cytB sequences of the larvae and one of the adults were identical and highly similar to those of the other adult and the deutonymph (which were identical; see Table 3), strongly supporting the conclusion that they belong to the same species. It is important to note here that we are studying only mitochondrial DNA, thus, in the case of hybrids, we would be only describing the maternal species of the larvae. In addition, the phylogenetic tree based on COI mitochondrial information (Figure 12) supports the idea that three *Arrenurus* subgenera are ‘natural’—*Arrenurus*, *Megaluracarus* and *Micruracarus*—and the somewhat arbitrary distinction of the species assigned to the subgenus *Truncaturus.* This topology differs from the only other *Arrenurus* phylogeny published [24] and points to the need for more molecular data in order to clarify their phylogenetic relationships.

This study demonstrates the usefulness of an integrative approach to resolve the taxonomic uncertainty of water mites at the larval stage. Furthermore, it appears to be a promising approach for identifying larvae at the species level in terms of both reliability and speed, in comparison with rearing or hatching approaches. With these data, we can also begin to elucidate host–parasite interactions between a specific water mite species and its host(s) with greater detail. To date, a host of *A. novus* larvae was unknown [2]. Indeed, only a few studies have described a host-parasite interaction between a *Culex* species and an *Arrenurus* species (e.g. [6,10,31,32]). *Arrenurus* is the genus most reported to parasitize mosquitoes, evidence of its flexibility regarding host specificity [33]. Despite this, recent findings suggest that these mites prefer *Culex* species [34]. Aside from reducing host fitness by piercing their exoskeleton to feed on hemolymph, each host–parasite association has its own characteristics, which are determined mainly by the size of the partners, intrinsic defence mechanisms and environmental conditions [35]. Parasitic mites may play a significant role in the biological control of mosquitos in wetlands, especially of adult populations. Therefore, more observations and experimental data of water mite species are needed to better understand their host–parasite interactions, as well as to incorporate the use of molecular techniques in their identification, particularly at the larval stage.

## Figures and Tables

**Table 1:** Standard measurements (see [8]) of the larval body morphology (in µm; only an approximation due to specimen orientation on the slide).

	L1	L2	L3	L4	L5
Body length	197	182	190	204	200
Body width	175	168	168	164	156
Dorsal plate length	190	186	190	185	185
Dorsal plate width	171	152	160	148	147
CpI medial margin length	57	55	61	57	62
CpII medial margin length	27	30	30	27	26
CpIII medial margin length	38	42	44	40	42
Distance between C1 and CpI median margin	21	19	23	18	20
Distance between C4 and CpIII median margin	28	27	24	27	29
Distance between C1 and C2	40	36	40	38	39
Excretory pore plate length	25	27	26	22	29
Excretory pore plate width	32	30	32	30	31
Distance between Exp and Expp posterior margin	11	15	15	12	13
PIII length	27	21	26	31	31
Length of PIV claw	27	23	14	16	17

**Table 2:** Standard measurements of the larval leg morphology in µm.

Larvae		Trochanter	Femur	Genu	Tibia	Tarsus
L1	leg1	25	21	29	42	55
	leg2	30	25	30	46	57
	leg3	30	25	30	46	57
L2	leg1	23	21	29	40	49
	leg2	23	25	30	44	59
	leg3	27	23	25	48	57
L3	leg1	30	23	30	38	49
	leg2	30	32	27	42	57
	leg3	34	34	27	44	57
L4	leg1	29	23	27	44	55
	leg2	21	25	29	46	53
	leg3	32	29	29	46	59
L5	leg1	24	23	27	40	41
	leg2	29	22	26	45	60
	leg3	30	25	30	40	31

**Table 3:** K2P distances among the adult, deutonymph and larval *Arrenurus* sp. cytB sequences analyzed in this study (alignment: 330 bp).

K2P	Adult 1	Adult 2	Nymph	Larvae
**Adult 1**	-			
**Adult 2**	0.006	-		
**Nymph**	0.006	0.000	-	
**Larvae**	0.000	0.006	0.006	-

## Supplementary Materials

The following are available online at https://www.mdpi.com/2075-1729/10/7/108/s1, Table S1: K2P distances among the COI sequences of the 52 *Arrenurus* species included in the analysis. The final alignment was 659 bp. *Arrenurus novus* is indicated in bold. Material S1: Maximum Likelihood phylogenetic tree of the *Arrenurus* COI sequences employed in this study and the outgroups (*Torrenticola lundbladi* and *Lebertia maderigena*) in newick format.
